# Severe vaginal bleeding due to vaginal metastasis from renal cell carcinoma with inferior vena cava tumor thrombus: A case report

**DOI:** 10.1097/MD.0000000000028586

**Published:** 2022-01-21

**Authors:** Zhihai Geng, Qinghua Zhang, Peng Jia, Jia Miao, Qian Lin

**Affiliations:** a Department of Urology, Taizhou First People's Hospital, Zhejiang Province, China.; b Department of Radiology and Interventional Medicine, Taizhou First People's Hospital, Zhejiang Province, China.

**Keywords:** bleeding, case report, inferior vena cava tumor thrombus, interventional embolization, renal cell carcinoma, vaginal metastasis

## Abstract

**Rationale:**

Renal cell carcinoma (RCC) is the most common type of kidney cancer and is the second most common urologic neoplasm. Vaginal metastasis from RCC is extremely rare clinically.

**Patient concerns:**

A 56-year-old woman presented with intermittent vaginal bleeding that had persisted for 1 month. Enhanced computed tomography examination suggested a vaginal mass (3 × 2 × 2 cm), right kidney tumor (15 × 12 × 10 cm), and an inferior vena cava tumor thrombus. During gynecologic examination, the mass was necrotic and caused uncontrollable vaginal bleeding.

**Diagnoses:**

Based on clinical and imaging examinations and the pathology, she was diagnosed as vaginal metastasis from RCC.

**Interventions:**

The patient received percutaneous transcatheter arterial embolization to stop uncontrollable vaginal bleeding, and then treated with targeted therapy.

**Outcomes:**

Vaginal bleeding disappeared after interventional embotherapy. However, disease progressed, and the patient died 9 months later.

**Lessons:**

In cases of vaginal bleeding, the possibility of metastatic renal cell carcinoma should be considered. Percutaneous transcatheter arterial embolization is an effective and novel treatment for uncontrollable vaginal bleeding caused by vaginal metastasis of RCC.

## 1 Introduction

Renal cell carcinoma (RCC) accounts for 3% to 5% of all adult malignancies worldwide and 80% of cancers involving the kidneys.^[1]^ RCC often metastasizes to the lungs and bones but rarely to the vagina. Vaginal metastasis from RCC is extremely rare, with fewer than 90 cases reported to our knowledge. Here, we present the case of a 56-year-old woman who presented with severe vaginal bleeding caused by vaginal metastasis of RCC. The uncontrolled vaginal bleeding was cured using interventional embolotherapy.

## 2 Case presentation

A 56-year-old woman presented to our department with a complaint of intermittent vaginal bleeding for 1 month. She had undergone left nephrectomy for severe left hydronephrosis 10 years ago and had no history of hypertension, diabetes, or hyperlipidemia. Her mother had a history of rectal cancer surgery.

Vital signs: *T* 37.2 °C, *P* 76 times/min, *R* 19 times/min, BP 118/62 mmHg. Abdominal physical examination revealed a wasted, anemic appearance with a flat abdomen, and no pressure or rebound pain. Gynecological examination revealed a vaginal mass located in the anterior vaginal wall, 3 cm from the vaginal opening, measuring approximately 3 cm × 2 cm × 2 cm.

Laboratory examination findings: HGB 87 g/L, Cr 144 μmol/L, BUN 8.9 mmol/L, ALB 34.8 g/L, UA 651 μmol/L, TCHO 2.79 mmol/L, PT 14.8S, ATPP 45.3S, D-Dimer 1.28 μg/mL.

**Figure 1:**
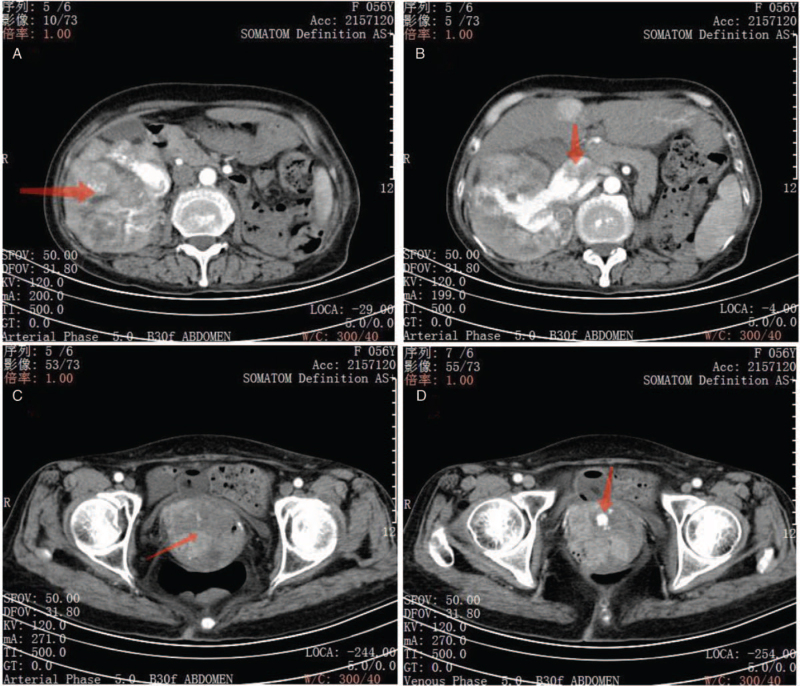
A, Abdominal enhancement CT shows irregular mixed hypodense mass in the right kidney with heterogeneous enhancement. B, Soft tissue mass visible in the vena cava with consideration of cancer embolism. C, Irregular vaginal mass with heterogeneous enhancement. D, Active bleeding within the vaginal mass with pseudoaneurysm formation. CT = computed tomography.

Enhanced computed tomography (CT) examination suggested a vaginal mass (measuring approximately 3 × 2 × 2 cm), right kidney tumor (measuring approximately 15 × 12 × 10 cm), and inferior vena cava tumor thrombus (Fig. 1). No abnormalities were observed on liver and biliary ultrasound, and no abnormalities were observed on chest and cranial CT.

**Figure 2:**
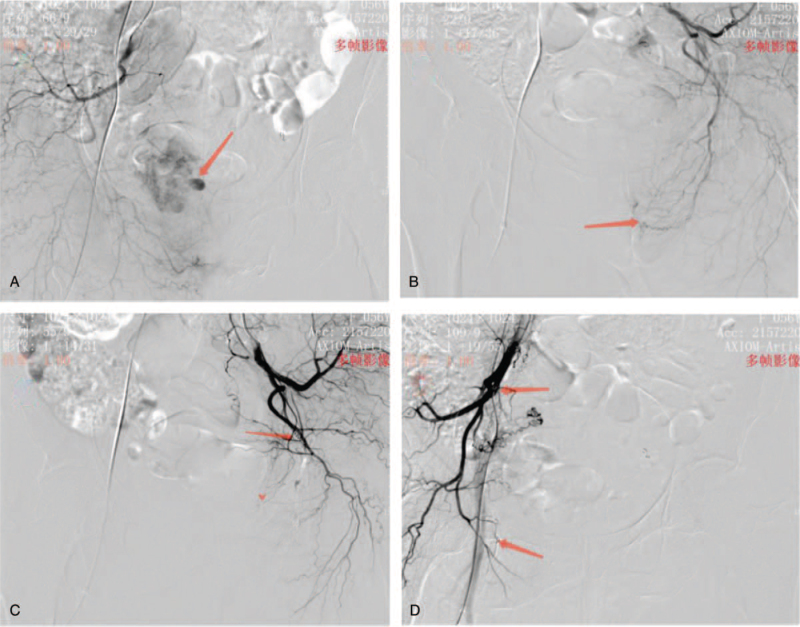
A, Pseudoaneurysm of the right uterine artery. B, Bleeding in the left internal pudental artery. C, After spring coil embolization of the left internal pudental artery. D, After spring coil embolization of the right uterine artery and internal pudental artery.

During gynecologic examination, the mass was necrotic and detached by itself with a hemorrhage of approximately 400 mL. Intravaginal gauze plugging was performed to stop bleeding, followed by selective bilateral internal iliac artery angiography. The angiogram showed that bleeding was predominantly on the right side. Embolization microspheres were used to embolize the right uterine artery and bilateral internal pudendal arteries, followed by embolization using spring coils (Fig. 2).

**Figure 3:**
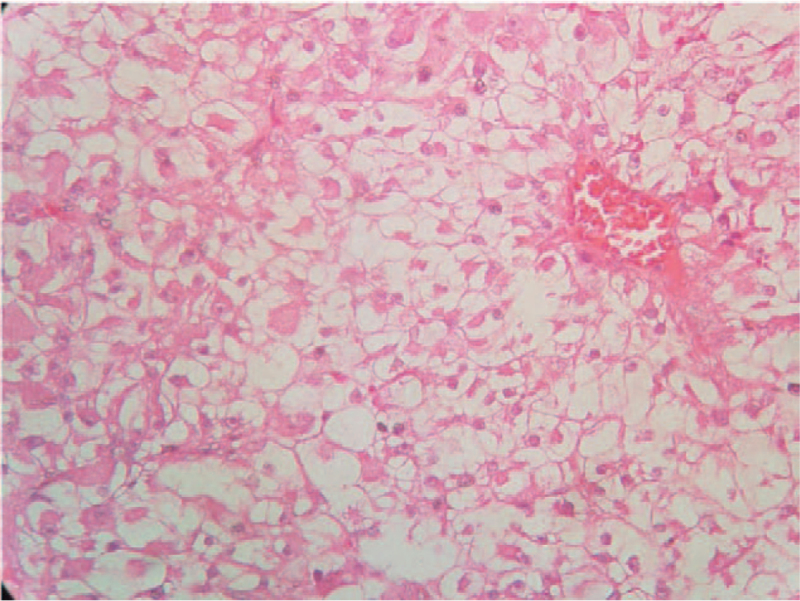
H. E. staining (400×), the tumor cells with nested, glandular vesicle-like arrangement, abundant and transparent cytoplasm, and obvious nucleol.

**Figure 4:**
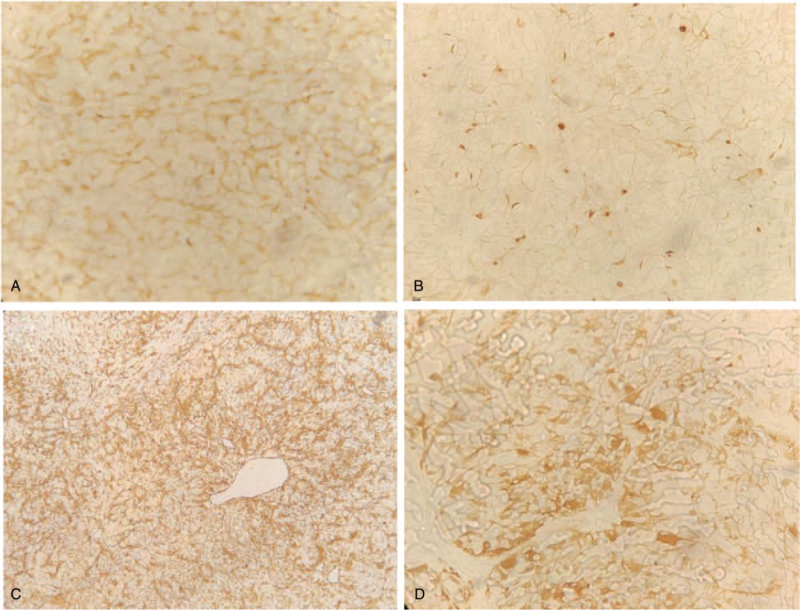
A, Immunohistochemically, the tumor cells were positive for CaIX in cell membrane. B, Immunohistochemically, the tumor cells were positive for CD10 in cytoplasmic/cytomembrane. C, Immunohistochemically, the tumor cells were positive for vimentin in cytoplasm positive. D, Immunohistochemically, the tumor cells were positive for CD8/18 in cytoplasm positive.

Pathology of the mass suggested clear cell carcinoma (Fig. 3). Immunohistochemical analysis revealed expression of CaIX, CD10, vimentin, and CD8/18 (Fig. 4). As she did not receive long-term hemodialysis, she refused further surgical treatment and received targeted therapy with sunitinib.

The vaginal bleeding disappeared after interventional embolotherapy using digital subtraction angiography. The disease progressed after 6 months of sunitinib treatment. For financial reasons, no other targeted drugs were used, and the patient died 3 months later due to renal failure.

## 3 Discussion

RCC is the most common type of kidney cancer and is the second most common urologic neoplasm. Synchronous metastasis occurs in 18% of patients with RCC at diagnosis and metachronous metastatic disease develop in 50% of RCC patients after nephrectomy.^[2]^ RCC metastasis can occur in any organ, most commonly in the lungs, lymph nodes, bones, and liver.^[3]^ Malignant tumors originating in the vagina are rare, and squamous cell carcinoma is the most common pathological type. Metastatic carcinoma accounts for approximately the majority of cases and should be prioritized in the absence of primary evidence. Common sites of vaginal metastasis reported by conventional investigations are the cervix, uterus, ovaries, colon, pancreas, and stomach. Vaginal metastasis from RCC is extremely rare, with fewer than 90 cases reported to our knowledge. Some patients experience vaginal bleeding as the first symptom. Therefore, the presence of vaginal bleeding needs to be evaluated to determine the presence of metastasis from RCC.^[4,5]^ The mechanism of metastasis of RCC to the vagina is still unknown, and the paths could include the bloodstream, lymphatic metastasis, urethral implant, and so on, with the bloodstream being the common route.^[6]^ Blood from the lower vaginal segment returns from the vaginal vein, forms a venous plexus with the uterine vein, converges into the internal iliac vein, and is injected into the inferior vena cava. Genital vein reflux is also a metastatic route for RCC, and the left side is directly refluxed to the renal vein; thus, vaginal metastasis of RCC is likely to occur on the left side.^[7]^ Mulcahy demonstrated retrograde flow of contrast medium from the renal vein to the genital vein in several patients with RCC and vaginal metastases.^[8]^

In this case, the patient's left kidney was resected 10 years ago, and CT imaging supported the diagnosis of right kidney cancer. Pathology suggested clear cell carcinoma, and immunohistochemistry revealed the expression of CaIX, CD10, vimentin, and CD8/18. These findings were consistent with the diagnosis of metastatic RCC. The incidence of vaginal metastatic cancer is increasing because of inferior vena cava tumor thrombus and impaired reflux. The vaginal blood supply comes from the internal iliac artery, and metastases are rich in blood supply and grow rapidly, making them prone to hemorrhagic necrosis and causing uncontrollable hemorrhage. Surgical treatment or radiotherapy is feasible for isolated vaginal metastatic lesions or local recurrence of renal cancer after surgery, surgical treatment, or radiotherapy is feasible.^[9,10]^ For cases with multiple metastases and advanced stages, where the chance of radical treatment is lost, systemic treatment and tumor reduction surgery are the mainstay.

In the patient, enhanced CT suggested a significant aneurysm blood supply, post-dissection hemorrhage, and pseudoaneurysm formation. Internal iliac arteriography using digital subtraction angiography can directly display the pseudoaneurysm and locate the bleeding site for subsequent embolotherapy. Percutaneous transcatheter arterial embolization is an effective and novel treatment for uncontrollable vaginal bleeding caused by vaginal metastasis of RCC. The median lifespan of a patient with RCC-associated vaginal metastasis is approximately 19 months.^[11]^ Further surgical treatment was refused because the patient had an isolated kidney and could not undergo long-term postoperative hemodialysis. The patient was treated with targeted sunitinib therapy. The patient's prognosis was poor due to the advanced stage of the disease at the time of detection.

## 4 Conclusion

Herein, we report a case of rare vaginal metastasis from RCC with severe vaginal bleeding as the first symptom. Percutaneous transcatheter arterial embolization is an effective and novel treatment for uncontrollable vaginal bleeding caused by vaginal metastasis of RCC. In cases of vaginal bleeding, we should have to keep in mind the possibility of metastatic renal cell carcinoma.

## Author contributions

**Conceptualization:** Qian Lin.

**Data curation:** Jia Miao.

**Investigation:** Qinghua Zhang.

**Methodology:** Peng Jia.

**Writing – original draft:** Zhihai Geng, Qian Lin.

**Writing – review & editing:** Zhihai Geng, Qian Lin.
